# Parental Feeding Practices in Families Experiencing Food Insecurity: A Scoping Review

**DOI:** 10.3390/ijerph19095604

**Published:** 2022-05-05

**Authors:** Kimberley A. Baxter, Smita Nambiar, Tsz Hei Jeffrey So, Danielle Gallegos, Rebecca Byrne

**Affiliations:** 1 Woolworths Centre for Childhood Nutrition Research, Faculty of Health, Queensland University of Technology, Graham St, South Brisbane 4101, Australia; smita.nambiar@qut.edu.au (S.N.); jeffrey.so@hdr.qut.edu.au (T.H.J.S.); danielle.gallegos@qut.edu.au (D.G.); ra.byrne@qut.edu.au (R.B.); 2 School of Exercise and Nutrition Sciences, Faculty of Health, Queensland University of Technology, Victoria Park Rd, Kelvin Grove 4059, Australia

**Keywords:** feeding practices, food insecurity, infant feeding, responsive feeding, parents, scoping review

## Abstract

Parental feeding practices and styles influence child diet quality and growth. The extent to which these factors have been assessed in the context of disadvantage, particularly household food insecurity (HFI), is unknown. This is important, as interventions designed to increase responsive practices and styles may not consider the unique needs of families with HFI. To address this gap, a scoping review of studies published from 1990 to July 2021 in three electronic databases was conducted. A priori inclusion criteria were, population: families with children aged 0–5 years experiencing food insecurity and/or disadvantage; concept: parental feeding practices/behaviours/style; and context: high income countries. The search identified 12,950 unique papers, 504 full-text articles were screened and 131 met the inclusion criteria. Almost all the studies (91%) were conducted in the United States with recruitment via existing programs for families on low incomes. Only 27 papers assessed feeding practices or styles in the context of HFI. Of the eleven interventions identified, two assessed the proportion of participants who were food insecure. More research is required in families outside of the United States, with an emphasis on comprehensive and valid measures of HFI and feeding practices. Intervention design should be sensitive to factors associated with poverty, including food insecurity.

## 1. Introduction

Parental feeding practices and styles play an important role in the development of child diet quality, eating behaviours and healthy growth [1]. Children are born with an innate ability to self-regulate their energy intake, which allows them to follow their own hunger and satiety cues [2]. This can be easily overridden by parental practices such as pressure to eat or the use of rewards for eating. These parent behaviours, referred to as ‘coercive control’ or non-responsive feeding practices, “teach” children to eat for reasons other than hunger [3]. Conversely, responsive feeding refers to prompt, emotionally supportive, contingent, and developmentally appropriate reciprocity between the child and their caregiver in relation to feeding and food intake [4]. Responsive practices fall broadly under the higher-order constructs of ‘structure’ and ‘autonomy support or promotion’ [3], whereby parents provide safe, nutritious, and developmentally appropriate foods and the child decides how much is eaten [1,5]. While practices are the specific goal-oriented actions a parent takes in relation to child feeding and eating, these sit within a broader construct known as feeding styles. Feeding style refers to the general way that parents interact with a child during meal and snack times [6]. An authoritative style is considered most appropriate, characterized by high levels of warmth and responsiveness to a child’s needs, along with high levels of age-appropriate reasoning and structure [7]. 

Over the last three decades, the global rise in the prevalence of childhood overweight and obesity prompted extensive research into the associations between parental feeding practices and styles with child outcomes. Responsive feeding practices are considered a protective factor in the prevention of excess weight and obesity [8,9], via the impact on a child’s ability to self-regulate their appetite and intake. Feeding practices also influence diet quality, for example, a pressure to eat has largely been associated with poorer quality diets in children, while parental modelling and encouragement are associated with improved diet quality, such as increased vegetable intake [10]. Such findings have led to the development of interventions aimed to modify feeding practices. Indeed, systematic reviews of randomized controlled trials of interventions found that promotion of responsive feeding is the most promising avenue for obesity prevention for children under two years [11,12]. However, exactly what components of interventions are most effective, and what components are most appropriate for different populations remains unclear [13]. This is particularly true for families experiencing socioeconomic disadvantage, who are disproportionally impacted by poor diet, suboptimal nutrition, and poor growth, including obesity [14]. 

Disadvantage, which includes financial and material hardship (low income, poor living conditions) and/or social isolation [15] has been strongly linked to poorer physical, cognitive, and social development in children [16]. The environmental conditions and adversity children experience during critical periods is known to impact on both immediate and long-term health. This has led to the nurturing framework linked to the sustainable development goals that posits that early child development is supported by seven key dimensions: good health, adequate nutrition, safety and security, responsive caregiving and learning and stimulation [17]. Within the context of responsive feeding, the nurturing framework is relevant; however, two circumstances may have particular significance for families living with disadvantage, that is, food insecurity and household chaos. Food insecurity is defined as the limited financial, physical, and social access to food of sufficient quality and quantity for a healthy and active life [18] and has been linked to poor child outcomes [19]. Food insecurity has a prevalence of around 12% at a population level in high income countries [20], with much higher rates in more disadvantaged communities. For example, in the USA 35.3% of households with incomes below the Federal poverty level were food insecure in 2020 [21], and in Australia up to 25% of households in low-income areas are affected [22,23]. A recent review of the literature by Gallegos et al. (2021) found that both persistent and transient household food insecurity were associated with sub-optimal child development outcomes [24]. Chaotic households that are prone to high noise and crowding, with low levels of routine, organisation and overall stability have been linked to poorer child development, overweight and obesity and food insecurity [25]. Household chaos and a lack of meal planning are potential mediating factors in food insecurity [26]. In contrast, responsive feeding is contingent on environments being pleasant, structured and without distractions, such that parents can recognize and respond to child cues in a prompt, developmentally appropriate way [4]. 

A narrative review by Arlinghaus and Laksa (2021) [27] argued that there are considerable structural constraints, such as the ability to access food and the cost of food, which influence how parents experiencing food insecurity feed their children. Those experiencing food insecurity have significantly more time constraints, particularly if they are single parents [27]. One of the benefits of responsive feeding, is that it promotes the development of healthy food preferences. Often, repeated exposure to novel foods is required before the child gains acceptance of a new food, but parents who are food insecure, may not offer foods that are not accepted immediately, particularly if they are expensive. The authors noted that low fruit and vegetable consumption may be the result of trying to prevent food wastage and the higher cost of such foods. 

Food insecurity can also be experienced intergenerationally, where chronic food insecurity shapes the way in which children learn about, acquire, and prepare food. There may be an emphasis on consuming foods with a high satiety value (that is, energy dense) over foods that are of higher quality (nutrient dense). Thus, interventions designed to support responsive feeding in households experiencing food insecurity, who may also have high levels of chaos, may require a different approach to commonly promoted strategies, such as repeated exposure to foods [28]. 

Therefore, the aim was to undertake a scoping review of the evidence related to parental feeding practices in families experiencing socioeconomic disadvantage—and food insecurity—in high income countries. The scoping review methodology was deemed appropriate to map the evidence and synthesise the key concepts given this diverse topic [29]. The objectives were to describe what and how parental feeding practices and styles have been assessed amongst families experiencing disadvantage, understand the characteristics of studies examining parent feeding practices in families with household food insecurity (HFI); and to identify and describe the key components of interventions that aim to modify feeding practices in families living with disadvantage and/or HFI. 

## 2. Materials and Methods

This review was compliant with the PRISMA checklist for scoping reviews [30] and the Joanna Briggs Institute (JBI) approach to scoping reviews [31]. The protocol was registered with the Open Science Framework (OSF) (doi:10.17605/OSF.IO/Q47VP) (created on 9 June 2021). 

### 2.1. Inclusion and Exclusion Criteria

A priori eligibility inclusion and exclusion criteria were developed as follows:

- Population: families with children aged 0–5 years experiencing HFI or disadvantage. Disadvantage could include a measure of HFI, poverty, low income, low education attainment, receiving welfare/food assistance or other indicators of socioeconomic disadvantage.
- Concept: Parental feeding practices or styles. Papers were included if a measure of parental feeding practices and/or styles was used or identified as a theme in qualitative research.
- Context: high income countries according to the World Bank definition [32].

Full-text, peer-reviewed articles that were published in English were included in this scoping review according to the above criteria between the years 1990 and 2021 (database searches conducted on 2 September 2020 and updated 12 July 2021). Articles were excluded if the population group had a diagnosed illness/disorder that would impact feeding (e.g., cystic fibrosis, premature birth), or the focus was on infant feeding practices exclusively (i.e., breastfeeding, use of formula, age of introduction of solid foods). Opinion pieces, editorials, reviews, conference abstracts or protocol papers were also excluded. 

### 2.2. Search Strategy

A search strategy was developed by KB and SNM in consultation with an experienced academic librarian. The search was run in three electronic bibliographic databases by KB (CINAHL, Medline and PsycInfo). Key words for the search strategy used in each database are shown in Appendix A. Citations were exported into EndNote and then imported into Covidence; a web based systematic review production tool [33]. The reference lists of included sources and relevant reviews were also checked.

### 2.3. Selection of Included Articles

The title and abstract of each article were screened in Covidence using *a priori* eligibility criteria. All authors were involved in the screening process. Two authors screened citations for inclusion independently, with inter-rater conflicts resolved by another reviewer, and this task was shared across authors (KB, SNM, RB, DG, JS). This process was repeated to screen full-text articles. The final list of included articles can be found in Appendix B. 

### 2.4. Data Extraction

Data extraction was completed in Covidence using a modified version of their data extraction form. Extraction was done by one author and checked by a second author for completeness. 

### 2.5. Data Synthesis and Analysis

Descriptive statistics were used to describe the characteristics of included papers, namely, those that directly measured and reported household food insecurity (HFI) using a specific tool and those that did not, country of origin, study design, and assessment of feeding styles or practices. The number of different feeding practices assessed across all papers were tallied, using the Vaughn content map of food parenting practices [3] as a guide and a count made of the most frequently used tools to assess styles and practices. 

Data from those papers that measured HFI were described in more detail including study design, primary objective, country of origin, sample characteristics (age, gender, recruitment details), measures and tools used and key findings. Similarly, a table describing intervention studies designed to modify feeding practices amongst families experiencing food insecurity was included. Given the search identified only two intervention studies with families that reported HFI, this table was expanded beyond the original objective, to also include interventions for families experiencing disadvantage. Findings were also synthesised descriptively to map the relevant aspects of the literature as related to our research question. Results of the review are presented in narrative form. Quality appraisal was not conducted as this was not deemed necessary to meet the objectives of the review. 

## 3. Results

**Figure 1:**
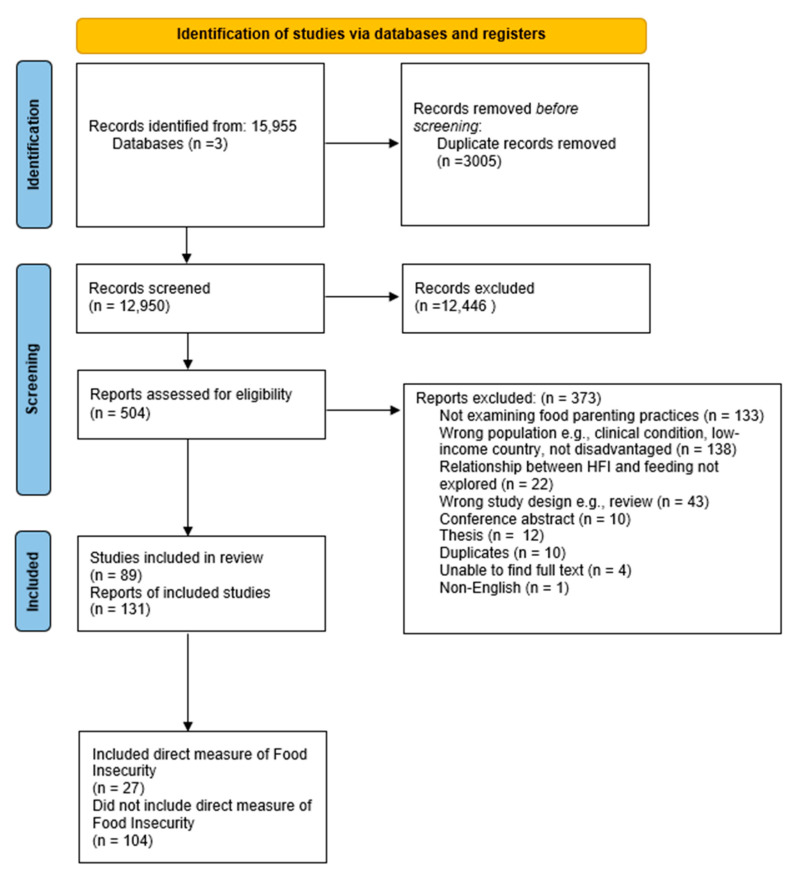
PRISMA diagram [38].

Searches identified 12,950 unique records (Figure 1). After screening, 131 met the inclusion criteria, with 27 studies (21%) assessing HFI within their population of interest (Table 1). Almost all studies were conducted in the United States (119/131, 91%) with the next most frequent location being Australia (6/131, 5%). 

**Figure 2:**
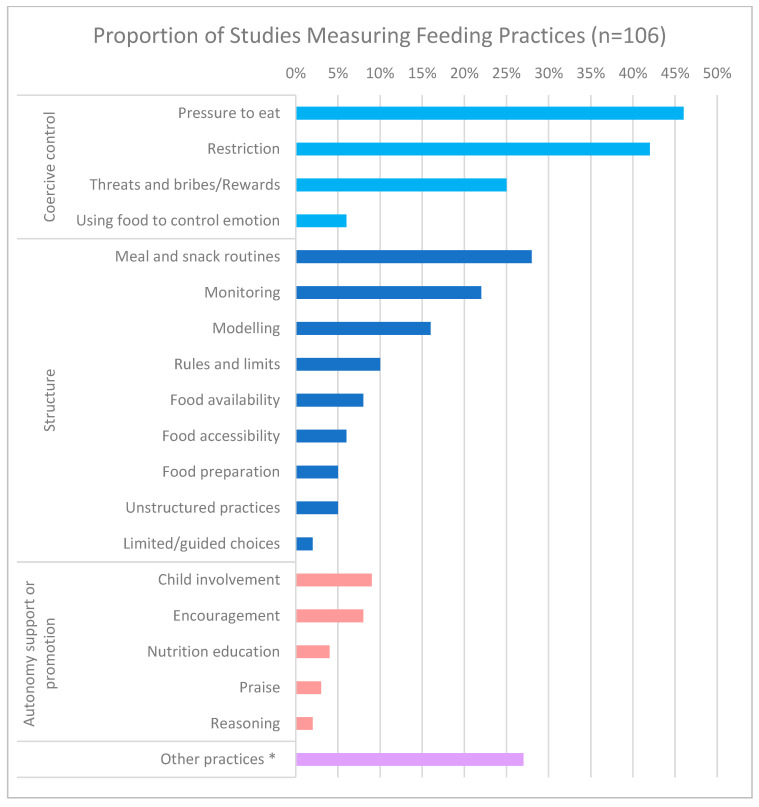
Proportion of studies measuring feeding practices (*n* = 106). * Representing a variety of disparate practices which do not fit strictly within the Vaughn framework.

One hundred and six papers examined feeding practices (81%). There was considerable heterogeneity in the types of practices assessed (Figure 2) and the tools used to assess these. Practices were categorised under the three higher-order food parenting constructs defined by Vaughn et al. (2016)—coercive control, structure, and autonomy support [3]. ‘Other’ practices included feeding practices that do not fall within the above known classification systems, such as laboratory eating protocols and food exposure practices.

Practices representative of coercive control such as a pressure to eat and restriction were most often assessed, in 46% and 42% of papers, respectively. Meal and snack routines were the most frequently assessed practice under the construct of ‘structure’ at 28% of studies, followed by the practice of modelling. Practices that aligned with ‘autonomy support and promotion’ were assessed least often. Another 29 studies (27%) were classified as other, representing a disparate set of practices that parents used to influence child intake or eating behaviour, but could not be easily categorised within the Vaughn framework. More than thirty different questionnaires were used to assess feeding practices within the studies included in this review, the most frequent being the *Child Feeding Questionnaire* (*n* = 26 studies) [34], followed by the *Comprehensive Feeding Practice Questionnaire* (*n* = 7) [35] and the *Feeding Practices and Structure Questionnaire* (*n* = 5) [36]. Forty papers assessed feeding styles within a population experiencing disadvantage, with the most used questionnaire being the *Caregiver Feeding Style Questionnaire* (CFSQ) [7] in 25 papers, while another 10 papers used the *Infant Feeding Style Questionnaire* (IFSQ) [37]. 

Validation studies identified in this review provide evidence that the psychometric properties of the *Child Feeding Questionnaire* (CFQ), *Caregiver’s Feeding Practices Questionnaire* (CFPQ) and the *Infant Feeding Style Questionnaire* (IFSQ) have been assessed in disadvantaged populations in the United States, in particular Hispanic and African American populations; however, no specific methodological studies assessing the use of tools outside of the US were found. 

### 3.1. Studies Examining Household Food Insecurity and Parental Feeding Practices/Styles 

The 27 papers identified are described in detail in Table 2. Twenty-three were conducted in the United States while the remaining four were in Australia. 

#### 3.1.1. Household Food Insecurity

In those studies that reported HFI (*n* = 27), a variety of tools were used to define HFI in their participant cohorts. Most studies (17/27, 63%) used a variation of the USDA Household Food Security Survey Module (HFSSM), namely, either the 6-item [39,40,41,42,43,44], 10-item [45,46,47], or 18-item measure [48,49,50,51,52,53,54,55]; followed by a 2-item measure by Hager et al. (2010) (3/27, 11%) [56,57,58] and a 1-item question from the Australian Health Survey (3/27, 11%) [59,60,61]. The Radimer/Cornell Scale was also used in one paper [62], along with the Household Food Insecurity Access Scale (HFIAS) in another one paper [63]. Lastly, the remaining two papers used less rigorous methods with one paper using a study specific question, ‘Do you ever feel that you don’t have enough food for your family?’ (no evidence of validity or reliability provided) [64] and one paper describing food insecurity as a theme from focus group discussions with low-income parents [65]. 

There was wide variation in the reported proportion of HFI experienced between the groups described in each of the papers, ranging between 0–80%. 

#### 3.1.2. Feeding Practices and/or Styles

The relationship between feeding practices and/or styles was most often examined within the context of child weight and obesity prevention [40,41,44,49,50,54,62,64]. The relationship between HFI and practices varied with HFI being associated with non-responsive practices in twelve [39,40,44,46,49,50,51,54,56,57,62,64] and non-responsive feeding styles in three [45,48,55] studies, respectively, with null findings in two others [41,64]. Interestingly, Kamdar et al. (2019), who found no relationship between feeding practices and styles, concluded that food insecurity may have a protective effect on dietary quality due to the adoption of coping mechanisms by mothers and grandmothers [41]. 

### 3.2. Intervention Studies to Modify Feeding Practices in Families Living with Disadvantage and/or HFI

Twelve studies described an intervention study that sought to modify early feeding practices amongst families who were categorised as low income, experiencing disadvantage and/or food insecure, these are summarised in Table 3. Only two of the interventions sought to assess and report the proportion of participants who were food insecure [50,58]. All the intervention studies identified originated from the US. Most of these research studies recruited participants via established programs for families on low incomes such as Head Start, Early Head Start or the Supplemental Nutrition Assistance Program (SNAP), with many research groups then utilising these existing programs and infrastructure to deliver the intervention. 

Length of the interventions ranged from a one-off video to three years (although the paper describing the 3-year intervention reported early outcomes at 10 months [50]). Interventions were largely aimed at mothers (10/12, 83% exclusively targeted mothers). Within one paper that included both mothers and fathers as participants, 92% were mothers [66] while the other paper reported participants as ‘parents’ and did not report the split of mothers to fathers [67]. 

Mode of delivery ranged from intensive multiple face-to-face appointments to remotely provided content via mail or phone and a computer tablet-based intervention in one case. Visual media content was a commonly used mode to deliver messaging in the interventions, with video described in several studies (*n* = 6, 50%) [58,68,69,70,71,72] as well as picture-based messaging [50]. In those papers using videos, these were described as short, curriculum-based videos, which included animation [72], real footage of mothers feeding their children in a home environment [68] and were tailored for the ethnicity of the target audience [68,69,70,72]. 

With the exception of Horodynski et al. 2005 [66], all the interventions described positive impacts on the intervention group in terms of the target feeding practices. Interventions largely targeted parental behaviours (feeding practices/styles), although Fisher et al. (2019) primary outcome was a reduction in calories from solid fat and added sugars (which was reduced by 23% at 12 weeks). Although many interventions had the underlying intention to prevent unhealthy weight gain among children, only Hughes et al. (2021) reported reduced child overweight/obesity compared to the control group [70]. Sun et al. (2017) showed a reduction in BMI among mothers in the intervention group compared with the control [72]. 

Of the two intervention papers that reported HFI, Fiks et al. (2017) found that HFI was significantly different at baseline between the intervention (HFI = 26%) and the control group (HFI = 60%) and, therefore, HFI was tested as a factor in their intention-to-treat analysis for health outcomes, with unchanged results. Messito et al. (2020) also reported the HFI rate of the participant cohort with 30.2% in the intervention and 34.5% in the control, which was found to be not significantly different at baseline. Messito et al. (2020) described tailoring content in the intervention to be sensitive to factors associated with poverty, including food insecurity [50]. 

**Table 2:** Details of studies examining feeding practices in families experiencing food insecurity (*n* = 27).

First Author, Date	Primary Objective	Country	Primary Recruitment Source	Child Details (Sample Size; Age Mean (SD) or Range; Sex; Weight Measure If Available)	Caregiver Details (Sample Size, Age Mean (SD); Relationship to Child; Ethnicity)	Degree of HFI	HFI Tool Used	Feeding Practice Tool	Key Outcome
**Quantitative**									
**Armstrong, et al. 2020 [39]**	To test associations among HFI, maternal restrained eating, and child feeding practices in low-income mothers of toddlers.	United States	SNAP for WIC and an urban paediatric clinic.	N = 277 20.11 (5.5) months 53% male BMI z-score 0.54 (SD1.13)	N = 277 27.28 (6.17) years Mothers African American (70%) Non-Hispanic White (8%)	40% food insecure	6-item USDA HFSSM [73]	TFBQ [74]	Relative increases in HFI were indirectly related to increases in restrictive and decreases in responsive child feeding practices, mediated through increases in mothers’ own restrained eating.
**Barroso et al. 2016 [40]**	To determine the association between measures of HFI, maternal feeding practices, maternal weight, and child weight-for-length in low-income Mexican Americans.	United States	WIC Clinics	N = 240 17 (4.17) months 51.7% male, 48.3% female healthy weight 47.1%, 52.9% overweight	N = 240 26.2 (5.81) years Mothers Hispanic (100%)	33% food insecure; 42% received SNAP	6-item USDA HFSSM [73]	CFQ [34] + study specific items	Children who were food insecure (SNAP recipients) were more likely to have a higher weight-for-length measurement.
**Berg et al. 2013 [63]**	To understand the relationships between parental perceptions about their child’s weight, feeding behaviours, acculturation, and HFI and obesity in childhood, in a low-income Hispanic population	United States	Three health fairs in a low-income Spanish speaking population	N = 85 3.24 (0.99) years underweight, 15.4%; healthy 41.7%; overweight, 21.4% obese, 21.4%	N = 85 30.91 years SD = 6.31 100% Hispanic	20% food insecure	The Household Food Insecurity Access Scale (HFIAS)—9 items [75]	CFQ [34]	Parents’ weight, perceptions of child’s weight, adherence to the Hispanic culture, and food insecurity appear to impact parental concerns and behaviours, particularly restrictive and pressure-to-eat behaviours.
**Fiks et al. 2017 [58]**	To examine the feasibility and acceptability of Grow2Gether (a peer group intervention delivered through Facebook) and to test the impact on behaviours	United States	Two high-volume, obstetric clinics (Medicaid insured)	9 months	N = 85 26.5 (5.4) years mothers 88% were black	42% food insecure	2-item household food security screener [76]	IFSQ—10 items [37]	A social media intervention resulted in high engagement and modestly improved feeding behaviours. Intervention reported significantly healthier feeding behaviours.
**Gross et al. 2018 [45]**	To determine the differential and additive impacts of HFI during the prenatal and infancy periods on obesity-promoting maternal infant feeding styles and practices at infant age 10 months.	United States	Secondary longitudinal analysis Details of recruitment NR	N = 412 10 months	N = 412 28.1 years mothers 100% Hispanics	39% food insecure	10-item USDA HFSSM [77]	IFSQ [37]	Prolonged HFI was associated with greater pressuring, indulgent and laissez-faire styles. Prenatal food insecurity was associated with less vegetable and more juice intake.
**Harris et al. 2018 [59]**	To examine the role of parent concern in explaining nonresponsive feeding practices in response to child fussy eating in socioeconomically disadvantaged families.	Australia	Socioeconomicaly disadvantaged urban community	N = 208 3.6 (1.0) years 50% female BMI-z score 0.67 (1.33)	N = 416 (i.e., 208 mother and father pairs) Mothers: 33.4 (5.3) years. Fathers 35.9 (6.6) years. ATSI (mother 4.8%, father 3.8%)	8% food insecure	1-item from Australian Health Survey [78]	FPSQ-28 [36]	In socioeconomically disadvantaged families, when parents are concordant in avoiding nonresponsive feeding practices, less child “food fussiness” is reported.
**Harris et al. 2019 [60]**	To examine if HFI modifies the relationship between child fussy eating and parents’ food provision and feeding with respect to exposure to a variety of healthy foods.	Australia	Socioeconomically disadvantaged urban community	N = 260 3.6 (1.1) years female 51% BMI z score 0.7 (1.3)	N = 260 33 (6) years mothers ATSI 5%	11% food insecure	1-item from Australian Health Survey [78]	FPSQ—1 item (36) + Food exposure practices [79]	Children’s fussy eating was associated with alternative meals in food insecure families. The availability of fruit was lower with HFI. Mothers’ food exposure practices may be contingent on the resources available.
**Horodynski et al. 2018 [48]**	To test the interactive effects of caregiver feeding style (CFS) and familial psychosocial risk in the association BMI-score in pre-schoolers from low-income families	United States	Head Start preschools	N = 626 48.99 months (6.13) girls (51%) BMI z-score Mean 0.62 (SD1.16)	N = 626 29.52 years (6.72) Primary caregivers non-Hispanic white (62%) and African American (30%)	37% food insecure	18-item USDA HFSSM [77]	CFSQ [7]	HFI was correlated with caregiver depressive symptoms and dysfunctional parenting. Uninvolved feeding styles intensified the risk, and an authoritative feeding style muted the risk conferred by living in a poor, food insecure and depressed family.
**Kamdar et al. 2019 [41]**	To investigate whether HFI affects child BMI through parental feeding demandingness and/or responsiveness and dietary quality 18 months later among low-income Hispanic pre-schoolers	United States	Head Start centres	N = 137 time point 1: 4.8 years; time point 2: 6.3 years 47.8% female normal 48.9%, overweight 21.2%, obese 29.2%	N = 137 dyads mothers, 2 grandmothers	46% food insecure	6-item USDA HFSSM [73]	CFSQ [7]	HFI had no influence on child BMI through feeding demandingness/responsiveness and/or child dietary quality. HFI was found to have a protective effect on dietary quality, this suggests the adoption of coping mechanisms
**McCurdy et al. 2014 [49]**	To examine why variation exists among child overweight in poor families with a focus on family food behaviours that are associated with income and maternal depression.	United States	Day care centres and a SNAP outreach project	N = 164 51.4 (10.1) months 55.5% male overweight (17.1%) obese (15.9%)	N = 164 30.1 (7.2) years mothers Hispanic (55%)	43% food insecure	18-item USDA HFSSM [77]	20 item FFBS [80]	Higher food resource management skills and greater maternal presence when the child ate was significantly associated with lower child BMI z-scores
**Melgar-Quiñonez et al. 2004 [62]**	To examine the relationship of child-feeding practices and other factors to overweight in low-income Mexican American preschool-aged children	United States	HeadStart; Healthy Start; SNAP; and migrant education programs.	N = 204 4.4 (0.8) years 51% female BMI: 17.0 (2.3)	N = 204 Age NR 50% mothers and 50% fathers Latino, Mexican American, Mexican, or Hispanic	80% food insecure	Radimer/Cornell scale (Spanish version) [81]	Control and autonomy support Survey (study specific items)	Variables positively associated with child overweight were income, mother’s BMI, child birth weight and juice intake. Biological and socioeconomic factors are more associated with overweight than self-reported child-feeding strategies.
**Messito et al. 2020 [50]**	To determine the impact of a primary care-based child obesity prevention intervention (StEP) beginning in pregnancy on maternal-infant feeding practices, knowledge, and styles at 10 months.	United States	Large urban public hospitals and affiliated health centres	N = 412 10.6 (0.7) month 48.5% male intervention 49.5% male control grp	N = 412 control: 28.8 (8.5) years intervention 28.9 (5.9) years mothers 100% female Hispanic	Control 70% food insecure Intervention 60% food insecure	18-item USDA HFSSM [77]	IFSQ 13 subscales [37]	StEP reduced obesity-promoting feeding practices and styles, and increased knowledge at 10 months. Integration into primary health care helped to reach high-risk families.
**Na et al. 2021 [51]**	To explore relationships between HFI, food resource management skills (FRM) and child feeding practices of low-income parents.	United States	Head Start preschools	N = 304	N = 304 32.2 (9.3) Non-Hispanic white (93.8%) 90% parent 95.4% Female	38% food insecure	18-item USDA HFSSM [77]	CFPQ [35]	Suboptimal child feeding is evident in low-income caregivers with low FRM skills,. Positive feeding practices were used by parents with high FRM skills regardless of HFI status.
**Orr et al. 2019 [56]**	To examine if caregiver feeding practices differed by household food security status in a diverse sample of infants.	United States	Paediatric clinics in academic teaching hospitals	N = 842 2.3 (0.4) months 51% female	N = 842 96% mothers, 4% father 28% black (non-Hispanic),18% white, 50% Hispanic, and 4% other.	43% food insecure.	2-item household food security screener [76]	IFSQ—15 items [37]	Feeding practices differed by HFI status. Food-insecure households had increased odds of agreeing with some obesity promoting practices such as immediately feeding a baby when they cry.
**Orr et al. 2020 [57]**	To examine associations between HFI status and parental feeding behaviour, weight perception, and child weight status in a diverse sample of young children	United States	Primary care paediatric residency training sites	N = 503 25 (1.3) months 49% Male, 51% Female	N = 503 52% Latino, 29% Black, 15% White, and 4% other.	37% food insecure	2-item household food security screener [76]	CFQ—31 items [34]	Parents with HFI reported more pressuring feeding behaviours and were more concerned about children becoming overweight.
**Perez et al. 2018 [52]**	To examine measurement equivalence of the CFQ and CEBQ across key contextual factors that influence paediatric obesity (gender, ethnicity, food security).	United States	paediatrician offices, day care centres, preschools, local shops or businesses frequented by families	N = 243 4.8 (0.85) years 51% male healthy 66.7%, overweight 23.8%, obese 9.5%	N = 243 70% mothers 33.6% Latino	30% food insecure	18-item USDA HFSSM [77]	CFQ 28 [34]	Both measures need continued psychometric work; group comparisons using some subscales should be interpreted cautiously. Subscales such as food responsiveness and restriction may be assessing behaviours that are less applicable in the context of HFI.
**Pesch et al. 2016 [53]**	To determine the association of child weight status with maternal pressuring or restricting eating prompts with four different types of food.	United States	Head Start	N = 222 70.9 months (8.53) 49.1% male normal weight 57.66%; overweight 22.07%, obese 20.27%	N = 222 White Non-Hispanic 73.42% mothers, or grandmothers	32% food insecure	18-item USDA HFSSM [77]	Structured eating protocol with BATMAN coding schema [82]	Mothers of children with obesity may alter their feeding behaviour differentially based on food type.
**Searle et al. 2020 [61]**	To examine associations between child temperament and parents’ structure-related feeding practices in a socioeconomically disadvantaged community.	Australia	Childcare centres, health clinic, family fun day, social media, newspaper	N = 205 3.6 years (1.0) 2–5 years 51% male	205 mother-father pairs ATSI 5%. 50% female 50% male	13% food insecure	1-item from Australian Health Survey [78]	FPSQ (three subscales) [36]	Perceptions of child food fussiness may explain why parents use less structure at mealtimes with children who have more difficult temperaments.
**Trappmann 2015 [64]**	To examine the relationship between HFI, childhood overweight, feeding behaviours, and use of federal public assistance programs among Head Start children from rural Hispanic and American Indian community.	United States	Head Start Centres	N = 374 47.71 months 97.73) 51% male BMI percentile 64.42 (26.91)	N = 374 77% mothers, 10% fathers, and 13% other caregivers Hispanic and Native American	21% food insecure	1 Item uncited question: Do you ever feel that you don’t have enough food for your family?	Control/pressure Study specific items	No significant relationships emerged between HFI and child overweight/obesity, certain feeding behaviours, or public food assistance utilisation. Further research is needed to understand these relationships.
**Zhou et al. 2020 [54]**	To test controlling parental feeding practices as mediating mechanisms by which child appetitive traits are linked to weight in an economically and ethnically diverse sample of children.	United States	Paediatricians’ offices, day care centres, preschools, local businesses.	N = 139 4.77 (0.84) years 51.8% male mean BMI: 16.47 (2.06)	N = 139 mothers 38.1% at or below the poverty line Hispanic 43.9%, European American 33.1%, African American 20.1%, Asian American 2.9%.	0% food insecure	18-item USDA HFSSM [77]	CFQ (pressure to eat and restriction subscales) [34]	Child appetitive traits are linked to child BMI through restrictive feeding or pressure to eat. Parents living in poverty endorsed higher levels of pressure to eat than those not in poverty.
**Qualitative**									
**Blaine et al. 2016 [42]**	To describe low-income pre-schoolers’ snacking and TV viewing habits, including social/physical snacking contexts, types of snacks and caregiver rationales for offering snacks.	United States	SNAP for WIC offices, playgrounds, Head Start centres and online	Target age = 3–5 years characteristics of children NR	N = 47 31.2(9.2) years 89% mothers 6% fathers 34% white, 34% African American, 32% Hispanic/Latino	47% food insecure	6-item USDA HFSSM [73]	Pressure; structure semi-structured interview	TV viewing and child snacking themes were consistent across racial groups. Caregivers facilitate snacking and TV viewing, which are described as routine, positive and useful.
**Davison et al. 2015 [55]**	To examine food parenting practices specific to child snacking among low-income caregivers.	United States	SNAP for WIC and online community listings such as craigslist	Target age = 3–5 years characteristics of children NR	N = 60 31.2 years (8.4) 92% mother, 5% father 30% non-Hispanic white, 37% African American, 33% Hispanic	43% food insecure	18-item USDA HFSSM [77]	control, structure, autonomy support, permissiveness. Semi-structured interview	Permissive feeding was added to the model. The conceptual model includes 4 feeding dimensions including autonomy support, coercive control, structure and permissiveness.
**Fisher et al. 2015 [43]**	To qualitatively describe low-income, urban mothers’ perceptions of feeding snacks to their preschool-aged children.	United States	SNAP for women, infants, and children (WIC)	51 months (37–66 months) female 47%	N = 32 27.5 years (20–41) mothers 91 % Black, 9% other, non-white	22% food insecure	6-item USDA HFSSM [73]	Structure and control Focus group	Mothers may perceive snacks as more important in managing children’s behaviour than providing nutrition. Snacks have a powerful hedonic appeal for mother and child.
**Gross et al. 2019 [46]**	To learn more about the financial pressures and perceived effects on infant and toddler feeding amongst low-income Hispanic mothers with children in infancy and toddlerhood.	United States	Large urban public hospital	N = 100 3 - 24 months old	N = 100 30 (6) years mothers 87% born outside of US 87% Spanish speaking 91% WIC participants	67% food insecure	10-item USDA HFSSM [77]	Restriction Semi-structured interview	HFI was frequently experienced, dynamic, complex and contributed to feeding beliefs, styles, and practices. Potential strategies—addressing misconceptions about maternal diet and breast milk, stress management, building social support, and connecting to assistance.
**Gross et al. 2021 [47]**	To understand how maternal stress, sadness, and isolation are perceived to affect feeding, to inform modifiable targets of interventions.	United States	large urban public hospital	N = 32 5.1 months (1.4) (3–7 months)	N = 32 29.3 years (6.6) Hispanic mothers	25% food insecure	10-item USDA HFSSM [77]	maternal-infant feeding interactions, laissez-faire, pressure to eat, infant emotions Interview	Maternal stress was perceived to negatively affect infant feeding. Mothers reported disrupting healthy feeding to avoid infant exposure to stress (including reduced breastfeeding).
**Herman et al. 2012 [44]**	To understand the contextual factors that influence how low-income mothers felt about addressing behavioural targets and mothers’ aspirations in child feeding.	United States	SNAP for WIC	N = 32 50.9 (36.9–65.9 months)47% female	N = 32 27.5 (20–41) years mothers 91% Black, 9% non-white	22% food insecure.	6-item USDA HFSSM [73]	Structure Focus group	Mothers’ aspirations in feeding were compatible with obesity prevention strategies to limit portion size and intake of fats/sugars. Mothers faced many feeding challenges.
**Tartaglia et al. 2021 [65]**	To explore parents’ experiences of feeding 0–5-year-old children and food literacy behaviours.	Australia	Parent-focused organisations in disadvantaged areas	N = 87 59.4% ≤ 2 years, 40.5% 3–5 years	N = 67 34 years (median) 92.5% parent, 4.5% grandparent, 3% guardian 92.5% female 22.4% ATSI	NR	HFI theme emerged from focus group discussion	Structure Focus group	Ten themes emerged and aligned with domains of relatedness, autonomy, and competence within self-determination theory. Parents were motivated to provide nutritious foods but faced many challenges.

NR = not reported; HFI = household food insecurity/insecure; FS = food security/secure; USDA HFSSM = United States Department of Agriculture Household Food Security Survey Module; SNAP = Special Supplemental Nutrition Program; BMI = body mass index; CEBQ = child eating behaviour questionnaire; WIC = women, infants, children. Feeding practice measurement tools: ATSI = Aboriginal or Torres Strait Islander; CFSQ = *Caregiver’s Feeding Style Questionnaire*; IFSQ = *Infant Feeding Style Questionnaire*; CFQ = *Child Feeding Questionnaire*; TFBQ = *Toddler Feeding Behaviour Questionnaire*; FPSQ = *Feeding Practices and Structure Questionnaire*; FFBS = *Family Food Behaviour Survey*; CFPQ = *Comprehensive Feeding Practice Questionnaire.*

**Table 3:** Studies describing an intervention to modify feeding practices amongst families living with HFI, low income or disadvantage (*n* = 12).

First Author, Date Name of INV Study Design	Description of Intervention	Length of INV	Mode of Delivery	Target Audience	Primary Outcome Measure/s	Tool Used	Results	Key Components
**Black, 1997 [68]** **“Feeding Your Baby with Love”** **RCT**	A video including messages, title, music, and setting were designed by an advisory group of 6 African American adolescent mothers who were filmed feeding their infants in their homes.	2 weeks	1 × 15-min video provided to participants to take home	N = 59 (INV = 26; Ctrl = 33) low-income, mothers 16.9 (1.3) years infants < 13 months 97% still in school 85% receive WIC African American	Attitudes toward feeding Maternal communication during mealtime At 2 weeks	About Your Child’s Eating (52-item questionnaire) [83] Parent–child interaction assessment [84]	INV mothers were more involved with their infant and reported more favourable attitudes toward feeding and communication	Culturally sensitive; adolescent mothers developed the vignettes and messages themselves, health professionals supported; realistic
**Fiks, 2017 [58]** **“Grow2Gether”** **RCT**	Private Facebook group INV commenced at 2 months prenatal until infant 9 months; video-based curriculum; foster behaviours promoting healthy parenting and infant growth. Moderated by a psychologist	11 months	Online social media group with short video curriculum posted weekly. Groups of 9–13 women	N = 87 (INV = 43; Ctrl = 44) low-income mothers 26.5 (5.4) years recruited when pregnant 42% food insecure Medicaid insured 80% African American	Maternal-infant feeding practices At 11 months	IFSQ—10 items [37]	INV reported significantly healthier infant feeding behaviours. INV mothers had higher healthy feeding behaviour scores; were less likely to pressure child to finish food. No differences in infant feeding beliefs or the timing of solids introduction.	Peer-group approach favoured by participants; high engagement (participants posted 30 times per group per week on average)
**Fisher, 2019 [85]** **“Food, Fun, and Families (FFF)”** **RCT**	Parenting INV aimed to reduce child consumption of empty calories from solid fat and added sugar (SoFAS). Content guided by authoritative food parenting theory; emphasised structure and autonomy support in feeding	12 weeks	12 in-person group sessions (60 min) of 8–12 mothers over 12 weeks Used behavioural change techniques e.g., goal setting and planning	N = 119 (INV = 59; Ctrl = 60) low-income mothers 29.8 (7.1) years children aged 3–5 years income qualified to receive SNAP 91% African American	Child measures: daily energy intake SoFAS post-test Authoritative food parenting practices At 12 weeks	24 h food recall Meal observations in a lab setting (study specific protocol)	FFF children consumed ~23% less daily energy from SoFAS than control group, adjusting for baseline levels. FFF mothers displayed a greater number of authoritative parenting practices when observed post-intervention.	FFF sessions were pilot tested with 9 women from a similar background.
**Horodynski, 2005 [86]** **“Nutrition Education aimed at Toddlers (NEAT)”** **Quasi-experimental**	Caregiver INV designed to improve caregiver-toddler mealtime interactions by empowering adults to become responsive to the child’s verbal and non-verbal behaviours	6 months	4 in-person group nutrition lessons (90 min) + 18 individual sessions (delivered by an EHS home visitor)	N = 135 (43 INV, 53 control) mean age 26 years (17–45), low-income mothers (92%); Caucasian (84%)	Child and parent mealtime behaviours At 6 months	Adapted child eating behaviour Inventory [87] The feeding self-efficacy questionnaire (8 items) (uncited)	INV showed higher knowledge scores. No statistically significant differences were found for measures of child and parent meal behaviours. Suggests looking at other avenues to enhance parents’ feeding practices.	After group sessions toddlers joined caregivers in food tasting, simple food preparation and family eating time.
**Hughes, 2020 [69]** **“Strategies for Effective Eating Development (SEEDS)”** **RCT** **Post Test Results**	Multicomponent family-based obesity prevention INV. Promotes self-regulation and healthy food preferences in low-income Hispanic children. Included parental strategies to promote appropriate portion sizes, structure, and routines, and dealing with outside influences on child eating. Curriculum informed by self-determination theory	7 weeks	7 in-person group lessons over 7 weeks. 8-10 mother–child dyads in each group. Videos and experiential learning activities reinforce the information.	N = 255 (136 INV and 119 control) 32.9 (6.8)–33.8 (7.3) years mothers children aged 3–5 years, children attending Head Start childcare Hispanic	Feeding knowledge/practices/styles (parent) BMI, eating self-regulation, trying new foods, fruit/vegetable consumption (child)	Parent: feeding knowledge survey, FPI [88], CFSQ [7] Child: compensation trials [89]; EAH [90], CEBQ [91]; willingness to try new foods (observation) [92,93] FPQ [94] weight (BMI)	Short-term post test results showed change in maternal feeding behaviours and knowledge, understanding feeding misconceptions and child roles in eating, and achieving feeding efficacy. Effects on child eating behaviour were minimal.	Experiential approach led to significant changes in behaviours; engagement was high, almost three quarters attended 5, 6, or all 7 of the lessons.
**Hughes, 2021 [70]** **“Strategies for Effective Eating Development (SEEDS)”** **RCT** **6- and 12-month results**	As above	7 weeks	As above	As above	As above	As above	INV had significant improvements in repeated exposure of new foods, measured portion sizes, child involvement in food prep, feeding responsiveness, knowledge of best feeding practices, and feeding efficacy, reduced feeding misconceptions and uninvolved feeding. Effects on child eating behaviour were minimal. At 12 months, children were less likely to be overweight/obese.	Outcome data at 6 and 12 months showed maintained improvement in key outcomes. Facilitators promoted a learner-based approach rather than a didactic one. Group session were pilot tested. Videos showed diversity
**Kugler, 2016 [95]** **Fractional factorial design**	Evaluation utilised multiphase optimisation strategy (MOST) to assess feasibility of a responsive parenting INV to prevent child obesity in low-income mothers with/without depression. Participants were randomised to 1 of 16 conditions using a factorial design with 8 components: responsive feeding (RF) (all participants), parenting, portion size, obesogenic risk assessment, mealtime routines, RF counselling, goal setting, mobile messaging, and social support	Length varied based on allocation Up to 4 weeks	INV was remotely delivered. RF and parenting curriculum (mail); portion size guidance (mail); obesogenic risk assessment (phone); personalised mealtime routine (phone); RF counselling (phone); social support (phone); mobile texts + videoes; Goal setting: (mail + phone)	N = 107 (*n* = 45) with and without (*n* = 62) depressive symptoms low-income mothers 29.2 years child aged 12 to 42 months participating in WIC 85% white, 8% Black, 5% Hispanic	Feasibility and acceptability of the intervention components and feasibility of implementing a factorial study design as part of a pilot study	Completion rates for each INV component; participant feedback on components (post-test interview)	Completion rates were high (85%) and did not statistically differ by depressive symptoms. All INV components were feasible to implement except for social support. Most participants reported the INV increased awareness of what, when, and how to feed their children. MOST provided an efficient way to assess the feasibility of components prior to testing with a fully powered experiment.	20% of participants receiving texts could not open the video messages sent INV primarily delivered by one research staff trained in health education
**Maher, 2010 [67]** **“Family Lifestyle Assessment of Initial Risk (FLAIR)”** **Qualitative study- content analysis**	A primary care obesity prevention INV targeting low-income minority parents. Identified family health risks and habits. Clinicians were trained in a patient-centred approach to deliver targeted brief behaviour change messages and set goals aligned with parents’ concerns.	NR	INV was delivered face to face alongside routine visits for paediatric patients. Supported by access to a health educator who provided brief behaviour change lifestyle counselling.	N = 83 low-income minority parents % mothers NR 92% Medicaid recipients child aged 24–59 months 26% of children were overweight/obese 80% Hispanic; 17% African American	Barriers to behaviour change experienced by families Strategies were to empower families to engage in healthy behaviour change.	Content analysis of health educator documents (FLAIR goal setting forms + action plans; clinical notes)	Themes were poor parenting skills (picky eating, food tantrums, bottle feeding, submitting to food requests), poor knowledge and skills regarding healthy eating, psychosocial issues (housing issues, parental unemployment, and intergenerational conflict regarding food choices).	A skilled, culturally competent, health educator is essential. Family focused approach. INVs need to be prepared for the degree of psychosocial difficulty that families face
**Messito, 2020 [50]** **“Starting Early Program (StEP)”** **RCT**	A primary care child obesity prevention INV for low-income, Hispanic families beginning in pregnancy through to child aged 3 years. Addressed feeding, activity, and general parenting.	3 years This paper reports at 10 months	Face-to-face individual nutrition counselling + nutrition and parent support groups coordinated with primary care visits. Content was developed for low health literacy, used picture-based messaging	N = 412 Low-income mothers control: 28.8 (8.5) years; INV: 28.9 (5.9) years food insecure 30% in INV; 34.5% Ctrl recruited in third trimester Hispanic families	Feeding styles Feeding practices (breastfeeding, introduction of cereal, water, and juice in the bottle and juice intake, self-feeding) At 10 months	IFSQ [37], Infant feeding practices study II [96]	INV showed greater breastfeeding, reduced juice and cereal in the bottle, and increased family meals than controls. INV had higher knowledge and lower nonresponsive feeding styles. High attendance at sessions.	Utilising primary care provided access to high-risk families; built on-existing provider relationships; reduced costs; saved time
**Moore, 2018 [71]** **Non-experimental pre-test post-test design**	A novel home-based motivational interviewing intervention to improve food parenting practices of low-income mothers with preschool-aged children. 5 food parenting practices: ‘pressure to eat’, ‘food as a reward’, ‘involvement’, ‘environment’, and ‘modelling’ were targeted	6 weeks	3 home face-to-face sessions approx. 2 weeks apart. At session 1 a family mealtime was videoed. Session 2 mothers watched segments of the video that included the targeted feeding practices to discuss and plan to improve these practices.	N = 15 mothers 32.3 (4.6) years child mean age = 3.2 years (0.9) low income Participate in WIC 86.7% white (mothers) 66.7% white (child)	Food parenting practices	5 subscales from the CFPQ [35] The Family Mealtime Coding System (video recorded meal) [97]	Mothers reported improvements in food parenting practices following the INV. INV had a decrease in controlling practices, ‘pressure to eat’ and ‘food as a reward’ and an increase in supportive practices, ‘involvement’, ‘environment’ and ‘modelling’. 93% of mothers ‘strongly agreed’ it was worth their effort to participate.	Most mothers found that watching themselves on video was informative and applicable to their own lives. Childcare was provided; INV conducted at times convenient to the mother
**Nix, 2021 [98]** **“Recipe 4 Success”** **RCT**	A preventive INV featuring structured food preparation lessons, designed to improve 4 protective factors related to overweight among families living in poverty: toddlers eating habits, toddlers’ self-regulation, parents responsive feeding practices, and parents sensitive scaffolding	10 weeks	10 face-to-face weekly home lessons as part of usual EHS visits. Lessons took ~45 mins. Focused on active coaching with structured food preparation activities using 3–6 ingredients. Toddlers could participate	N = 73 mothers child aged 30.72 months (6.96) months low-income families enrolled in Early Head Start 78% SNAP recipients 48% non-Hispanic white; 29% Black; and 23% Hispanic/Latino	Child: healthy eating habits; self-regulation Mother: responsive feeding practices [9] and sensitive scaffolding [99]	Child: 24-h food recall; snack delay task [100]; infant behaviour record [101]; infant-toddler social and emotional assessment [102] Video recordings of (1) parent introducing new foods and (2) 3 × 3 min interaction tasks	INV toddlers consumed healthier meals/snacks and displayed better self-regulation. INV parents were more responsive and were better able to sensitively scaffold their toddlers’ learning and development. Showed medium to large INV effects on the 4 protective factors that are often compromised by living in poverty.	Cocreated by administrators and home visitors from EHS. Used the pre-existing infrastructure of EHS for INV dissemination. Ingredients for the food preparation supplied
**Sun, 2017 [72]** **RCT pilot**	A family-centred, technology-based INV to improve health behaviours of low-income, overweight/obese Chinese mothers and their children. Guided by the Information Motivation Behavioural Skills Model. The INV used images, food items, and sample menus familiar to the Chinese culture.	8 weeks	8 weekly 30-min, interactive, Cantonese sessions accessed via table computers. 6 lessons were10 to 15-min animated videos; 2 lessons were a talk show format hosted by a bicultural dietitian with Cantonese speaking mothers	N = 32 low-income Chinese mothers with low acculturation; basic computer/internet skills Head start participants 36 (4.9) years child aged 4.31 (0.69) years Chinese	Maternal outcomes: self-efficacy, eating behaviours, physical activity, child-feeding practices, and BMI At 3 and 6 months	CFQ-28 [34] The Family Eating and Activity Habits Questionnaire [103] Maternal Self-Efficacy 12-item scale (uncited)	The INV was feasible. Significantly more INV mothers decreased BMI and increased their confidence for promoting healthful eating at home compared to control. Other outcomes saw small to medium improvement. There was no difference in child BMI.	Tailored content. INV was adapted from previous research. Tablet provided by the INV INV created a theme song with key messages that mothers could sing to their child

INV = intervention; RCT = randomised controlled trial; HFI = household food insecurity; CI = confidence interval; EHS = Early Head Start. Tools/measures: CFQ = *Child Feeding Questionnaire*; CFPQ = *Caregiver’s Feeding Practices Questionnaire*; IFSQ = *Infant Feeding Style Questionnaire*; CFSQ = *Caregivers Feeding Styles Questionnaire*; CEBQ = *Children’s Eating Behaviour Questionnaire*; FKQ = *Feeding Knowledge Questionnaire*; FPI = *Food Parenting Inventory*; FPQ = *Food Preferences Questionnaire*; EAH = eating in the absence of hunger protocol.

## 4. Discussion

This scoping review examined the evidence related to parental feeding practices and styles in families with a young child (aged 0–5 years) experiencing socioeconomic disadvantage (with and without food insecurity)—in high income countries. After using broad search terms of socioeconomic disadvantage, of the 131 papers identified, only 27 (21%) papers were found to address the issue of household food insecurity (HFI), and only two of these papers described an intervention to support responsive feeding in families experiencing HFI. Whilst the evidence on the direct impact of food insecurity on parental feeding practices is scant, the literature suggests that it does likely influence how and what parents feed their children. Parental feeding practices are sensitive to factors which influence the feeding environment such as food insecurity and, therefore, such factors are important to consider in parental feeding practice research and intervention design.

This review identified the most common measures used to assess feeding practices and styles, though there was little evidence that the validity and reliability of these tools have been assessed amongst families experiencing HFI. The practices most frequently assessed—pressure to eat and restriction—fall within the higher order construct known as ‘coercive control’, while fewer studies assessed ‘structure’ related feeding practices. In the future, studies could assess the aspects of structure to better elucidate the relationship between HFI, household chaos and a family’s ability to implement responsive feeding practices. Very few papers examined practices related to ‘autonomy support or promotion’. While the reasons for this cannot be determined from the review, it may be that practices such as educating children about the benefits of healthy eating or child involvement in meal planning and preparation may be considered less applicable in children under the age of five years. 

Variation in the tools used to measure HFI makes describing and comparing HFI amongst populations challenging and there are calls for greater consistency in measuring food insecurity [24,104]. This was reflected in this review, which found significant variation in the measures used to describe HFI. Several studies used short 1- or 2- item measures (7/27, 36%). Whilst these measures provide an indication of HFI levels, they may be less reliable and may also underestimate HFI by 5–8% points when compared to more rigorous, multi-item tools [104,105]. The most used HFI measure was the 18-item United States Department of Agriculture Household Food Security Survey Module (USDA HFSSM), which was the predominant tool cited in the literature [105,106]. The 18-item USDA HFSSM includes eight child-related items and therefore may be the most relevant in the context of parental feeding practices and HFI research which focuses on child-related outcomes. In this review 8/27, 30% of the papers used the 18-item USDA HFSSM which includes the child specific items. The short form (6-item) and 10-item form USDA HFSSM were also found to be used among 9/27 (33%) of the included papers. Studies balance the burden of administering tools and surveys to their participant group and therefore may opt for shorter measures of HFI; however, choosing measures that account for HFI severity and allow for child specific measures may be advantageous in parenting feeding practice research, especially in the context of socioeconomic disadvantage where the prevalence of HFI is likely to be high. In addition, the degree of severity of HFI may influence the type and frequency of feeding practices used at any given time. 

Another strong feature of the parental feeding practices and socioeconomic disadvantage/HFI literature summarised here is the heavy representation of US populations, which commonly draw on Head Start/Early Head Start and SNAP programs for recruitment. Studies conducted in the United States also tend to have a high proportion of Hispanic, Latina and/or African American participants. Perceptions of ideal body size, appropriate meal-time practices and family traditions vary across culture, and conceptualisations of “ideal” feeding practices in the scientific literature may clash with culture and community [107]. This may reduce the applicability of research findings to other countries or social and government assistance contexts outside of the US. Given that high-income countries, outside of the US, have evidence of significant HFI among their population, particularly in disadvantaged groups, this is of note and indicates the need for further research into HFI in other high-income countries. Whereas the US has readily identifiable groups among their population to recruit for research purposes (e.g., SNAP and Head Start), recruitment for such studies can be challenging in other countries due to the difficulty in identifying and successfully recruiting socioeconomically disadvantaged groups. In addition, food insecurity is monitored annually in the USA and has been identified as a significant public health issue, thus potentially highlighting it as an area of concern [108]. Further research may therefore also be warranted identifying successful avenues to recruit disadvantaged and HFI groups, which may also facilitate further research in this area. 

A recent narrative review of parent feeding practices in the context of food insecurity identified no existing interventions that target parent feeding practices specifically addressing the context of food insecurity [27]. Our scoping review of the literature supports this finding and whilst two interventions were identified which reported HFI, only one of those appeared to take into account the poverty related challenges of food insecurity [50]. This review adds to the evidence by identifying some of the key features and characteristics of interventions targeting feeding practices in disadvantaged groups. The intervention studies identified in this review showed largely positive improvements in the parent and child outcomes measured subsequent to participation in the intervention. 

A key feature identified in the interventions summarised was the high use of visual media content. Video and/or images are often used to convey messages to low health literacy groups. A systematic review has identified that pictorial information improves understanding and recall and is most impactful in the lowest health literacy groups [109]. Black and Teti (1997) developed a video which featured mothers from their target population, i.e., low-income adolescent African American mothers [68]. The video content, messaging and music was developed by an advisory panel of six African American adolescent mothers who were featured in the footage in their own homes feeding their babies. This culturally sensitive approach enhances the relatability of the messages. Other studies also adapted intervention content for their specific audience, including Sun et al. (2017) who developed an intervention for Chinese immigrant mothers and included videos in Cantonese featuring Chinese mothers with their children, including images, sample menus and foods which were also tailored to the Chinese culture [72]. Hughes et al. (2021) reporting on the intervention, ‘Strategies for Effective Eating Development (SEEDS)’, also utilised short videos in their face-to-face group sessions [70]. Videos can also be used in interventions to moderate the content and direct the conversation to targeted positive parent behaviours, such as in the ‘Grow2Gether’ intervention by Fiks et al. (2017)—an online social media group-based intervention that encouraged participation and discussion among peer mothers [58]. Videos were posted on closed social media groups, which acted to deliver positive feeding messages as well as to be a catalyst for productive discussion among participants around the content. Short, realistic, and relatable videos and media may be a successful feature to incorporate into interventions targeting parents from low income, disadvantaged backgrounds.

The summarised interventions also demonstrated that a range of modes of delivery can be successful in this group, including traditional approaches of intensive face-to-face individual or group delivery of nutrition-based information, to remote modes of intervention delivery (i.e., video, mailed content, social media, and technology-based interventions). This is important given the context of COVID-19 impacting health service delivery and the engagement with families of young children [110]. Traditional, intensive, face-to-face interventions may not be practical or feasible in a post-COVID-19 environment and it may take some time until families are willing or able to attend such intensive face-to-face interventions. It is also important to note that the one intervention that showed no positive impact on parent behavior, Horodynski et al. (2005), was the most intensive of the interventions described with 4 group sessions and 18 individual home visits over 6 months [66]. This suggests that interventions need to move beyond intensive face-to-face sessions and instead implement multi-modal strategies to engage families.

This scoping review also identified aspects from the summarised papers that reported HFI (*n* = 27) that may be potential areas to explore or target in interventions. Some of the studies highlighted different strengths within families that could potentially protect parental feeding practices from the negative impact of HFI. Food resource management (FRM) skills is one area that could be further explored. McCurdy et al. (2014) showed that better FRM skills and parental presence at meals was associated with healthier weight among 2–5-year-old children in low-income families. The potential pathway between FRM skills and healthier child weight needs to be further elucidated, but the mechanisms suggested by McCurdy et al. (2014) may reduce takeaway consumption due to more home cooking, parent modelling of healthy eating, as well as an increased structure in feeding practices, e.g., more family meals and parent presence at mealtimes. The potential role of FRM skills was also described in Na et al. (2021), which reported that low FRM skills were associated with suboptimal child feeding with and without HFI. In this paper, parents in food insecure households who had high FRM skills used similarly positive feeding practices as parents from food secure households with high FRM skills [51]. Kamdar et al. (2019) also suggests that families may use coping strategies which may mitigate the negative consequences of HFI. This paper found that dietary quality improved over 18 months in HFI families which was unexpected and needs further research but may indicate the adoption of coping strategies among families [41]. These findings, although requiring further exploration and research, may suggest how interventions can be designed to incorporate the strategies and coping mechanisms families who are at high risk of HFI already use to mitigate the negative impact of HFI on their feeding practice. 

It is also important to note that all the interventions identified within this review focused on individual behaviour change strategies, particularly that of mothers. This approach has been criticised for placing the responsibility for a child’s health solely on the mother and failing to advocate for structural interventions (e.g., policy change) to support parent feeding practices [111]. Researchers and practitioners are encouraged to utilise a socioecological model to intervene across systems for maximum impact [24].

This review has several strengths. It followed best practice guidelines using an a priori protocol. Due to the inconsistency of terminology used in the literature to describe feeding practices and styles, a deliberate decision was made to use broad search terms to identify as many papers as possible; however, given that some included studies (e.g., qualitative studies employing interview or focus group methodologies) did not set out to assess or describe HFI and feeding practices or styles, but these issues were raised by participants and reported in the results, it is possible that similar papers were not identified and included. This should be considered as a limitation.

## 5. Conclusions

This scoping review highlights the lack of research at the crossover of parental feeding practices and food insecurity, especially in terms of interventions that target feeding practices among groups likely to have a high prevalence of food insecurity. More research is needed outside of the United States, with an emphasis on comprehensive and valid measures of HFI and feeding practices. Intervention design should be sensitive to factors associated with poverty, including food insecurity.

## Figures and Tables

**Table 1:** Summary of studies examining feeding practices and/or styles amongst families experiencing disadvantage, including food insecurity (N = 131).

Study Characteristic		% (N)
Target population - Food Insecure - Low income/other measure of disadvantage		21% (27) 79% (104)
Country of Origin - United States of America - Australia - United Kingdom - Germany - Chile		91% (119) 5% (6) 3% (4) 1% (1) 1% (1)
Feeding style examined		31% (40)
Feeding practices examined		81% (106)
Type of Study Design		
Quantitative	Cross sectional * Longitudinal Intervention Validation	43% (56) 11% (15) 8% (11) 7% (9)
Qualitative	Interview Focus Group Discussion Content Analysis of an Intervention Longitudinal	11% (14) 12% (16) 1% (1) 1% (1)
Mixed Methods Design		6% (8)

* Includes studies using direct observation of parent–child dyads, using a coding schema to quantify practices.

## Data Availability

Not applicable.

## Appendix A

**Table A1:** Key words for the search strategy used in each database.

Database/ Platform	Term 1 Parent/Child	Term 2 Feeding	Term 3 Food Insecurity
Medline (via EBSCOhost)	Keyword: Parent * Mother * maternal caregiver * father * Child * Infant * baby MeSH: child father-child relations father mother-child relations parents mothers maternal behaviour parent-child Relations	Keyword: Feeding “Complementary feeding” weaning “eating behaviour” “food preferences” MeSH: feeding methods infant food eating weaning food preferences	Keyword: “Food insecur *” “Food secur *” “Food shortage” “Food scarcity” “Food supply” Hunger “low income” poverty disadvantage * “food insufficiency” “low resource household” MeSH: Food Assistance Social security Food deprivation Working poor Health equity poverty
PsychInfo	Keyword: As above PsychInfo Thesaurus: Mothers Mother Child Communication Family Relations Father Child relations Father child communication Mother child relations Parent child relations Parental role Parents parental attitudes Parental characteristics	Keyword: As above PsychInfo Thesaurus: Food Eating behavior Weaning Mealtimes Food Intake Food preferences	Keyword: As above PsychInfo Thesaurus: Disadvantaged Economic Disadvantage Food Insecurity socioeconomic factors economic inequality poverty lower income level Hunger social issues social disadvantage socioeconomic status Family socioeconomic level economic resources food deprivation
CINAHL	Keyword: Parent * mother * Maternal Caregiver * Father * Child * Infant * baby CINAHL Terms: child father-child relations father+ mother-child relations parents+ mothers+ maternal behaviour parent-child Relations+ MM Infant Father-infant relations Mother-infant relations Parent-infant relations	Keyword: Feeding “Complementary feeding” weaning “eating behaviour” “food preferences” CINAHL Terms: MM feeding methods infant food eating weaning food preferences Child Nutritional Physiology Infant Nutritional Physiology infant feeding eating behavior	Keyword: “Food insecur *” “Food secur *” “Food shortage” “Food scarcity” “Food supply” Hunger “low income” poverty disadvantage * “food insufficiency” “low resource household” “working poor” CINAHL Terms: Food Assistance Food Security Economic and Social Security Poverty Poverty areas

## Appendix B. List of Included Papers in the Scoping Review

1. Agrawal T, Farrell TJ, Wethington E, Devine CM. “Doing our best to keep a routine:” How low-income mothers manage child feeding with unpredictable work and family schedules. Appetite. 2018;120:57–66.
2. Anderson CB, Hughes SO, Fisher JO, Nicklas TA. Cross-cultural equivalence of feeding beliefs and practices: the psychometric properties of the child feeding questionnaire among Blacks and Hispanics. Preventive medicine. 2005;41(2):521–531.
3. Arlinghaus KR, Hernandez DC, Eagleton SG, Chen T-A, Power TG, Hughes SO. Exploratory factor analysis of The Comprehensive Feeding Practices Questionnaire (CFPQ) in a low-income hispanic sample of preschool aged children. Appetite. 2019;136:N.PAG-N.PAG.
4. Arlinghaus KR, Vollrath K, Hernandez DC, Momin SR, O’Connor TM, Power TG, et al. Authoritative parent feeding style is associated with better child dietary quality at dinner among low-income minority families. The American journal of clinical nutrition. 2018;108(4):730–736.
5. Armstrong B, Hepworth AD, Black MM. Hunger in the household: Food insecurity and associations with maternal eating and toddler feeding. Pediatric obesity. 2020:e12637.
6. Barrett KJ, Thompson AL, Bentley ME. The influence of maternal psychosocial characteristics on infant feeding styles. Appetite. 2016;103:396–402.
7. Barroso CS, Roncancio A, Moramarco MW, Hinojosa MB, Davila YR, Mendias E, et al. Food security, maternal feeding practices and child weight-for-length. Applied nursing research: ANR. 2016;29:31–36.
8. Bauer KW, Haines J, Miller AL, Rosenblum K, Appugliese DP, Lumeng JC, et al. Maternal restrictive feeding and eating in the absence of hunger among toddlers: a cohort study. The international journal of behavioral nutrition and physical activity. 2017;14(1):172.
9. Baughcum AE, Burklow KA, Deeks CM, Powers SW, Whitaker RC. Maternal feeding practices and childhood obesity: a focus group study of low-income mothers. Archives of pediatrics & adolescent medicine. 1998;152(10):1010–1014.
10. Baughcum AE, Powers SW, Johnson SB, Chamberlin LA, Deeks CM, Jain A, et al. Maternal feeding practices and beliefs and their relationships to overweight in early childhood. Journal of developmental and behavioral pediatrics: JDBP. 2001;22(6):391–408.
11. Beck AL, Hoeft KS, Takayama JI, Barker JC. Beliefs and practices regarding solid food introduction among Latino parents in Northern California. Appetite. 2018;120:381–387.
12. Bekelman TA, Bellows LL, Clark L, Thompson DA, Kemper G, McCloskey ML, et al. An Ecocultural Perspective on Eating-Related Routines Among Low-Income Families With Preschool-Aged Children. Qualitative health research. 2019;29(9):1345–57.
13. Berg J, Tiso S, Grasska M, Tan E, Chowdhury Y, Zender R, et al. Obesity, Parent Perceptions, Child Feeding, and Food Security in First Generation Hispanic Families. Californian Journal of Health Promotion. 2013;11(3):86–92.
14. Berge JM, Miller J, Veblen-Mortenson S, Kunin-Batson A, Sherwood NE, French SA. A Bidirectional Analysis of Feeding Practices and Eating Behaviors in Parent/Child Dyads from Low-Income and Minority Households. The Journal of pediatrics. 2020;221:93.
15. Black MM, Teti LO. Promoting mealtime communication between adolescent mothers and their infants through videotape. Pediatrics. 1997;99(3):432–437.
16. Blaine RE, Fisher JO, Blake CE, Orloski A, Younginer N, Bruton Y, et al. Conditioned to eat while watching television? Low-income caregivers’ perspectives on the role of snacking and television viewing among pre-schoolers. Public health nutrition. 2016;19(9):1598–1605.
17. Branscum P, Lora KR. Development and Validation of an Instrument Measuring Theory-Based Determinants of Monitoring Obesogenic Behaviors of Pre-Schoolers among Hispanic Mothers. International journal of environmental research and public health. 2016;13(6).
18. Cartagena D, McGrath JM, Linares AM. Associations between Introduction of Age-Inappropriate Foods and Early Eating Environments in Low-Socioeconomic Hispanic Infants. Journal of pediatric health care: official publication of National Association of Pediatric Nurse Associates & Practitioners. 32(2):e27–e36.
19. Corbett KS. Explaining infant feeding style of low-income black women. Journal of pediatric nursing. 2000;15(2):73–81.
20. Cross MB, Hallett AM, Ledoux TA, O’Connor DP, Hughes SO. Effects of children’s self-regulation of eating on parental feeding practices and child weight. Appetite. 2014;81:76–83.
21. Davison KK, Blake CE, Blaine RE, Younginer NA, Orloski A, Hamtil HA, et al. Parenting around child snacking: development of a theoretically-guided, empirically informed conceptual model. The international journal of behavioral nutrition and physical activity. 2015;12:109.
22. Elias CV, Power TG, Beck AE, Goodell LS, Johnson SL, Papaioannou MA, et al. Depressive Symptoms and Perceptions of Child Difficulty Are Associated with Less Responsive Feeding Behaviors in an Observational Study of Low-Income Mothers. Childhood obesity (Print). 2016;12(6):418–425.
23. Fiks AG, Gruver RS, Bishop-Gilyard CT, Shults J, Virudachalam S, Suh AW, et al. A Social Media Peer Group for Mothers To Prevent Obesity from Infancy: The Grow2Gether Randomized Trial. Childhood obesity (Print). 2017;13(5):356–368.
24. Fisher JO, Serrano EL, Foster GD, Hart CN, Davey A, Bruton YP, et al. Title: efficacy of a food parenting intervention for mothers with low income to reduce preschooler’s solid fat and added sugar intakes: a randomized controlled trial. The international journal of behavioral nutrition and physical activity. 2019;16(1):6.
25. Fisher JO, Wright G, Herman AN, Malhotra K, Serrano EL, Foster GD, et al. “Snacks are not food”. Low-income, urban mothers’ perceptions of feeding snacks to their preschool-aged children. Appetite. 2015;84:61–67.
26. Galindo L, Power TG, Beck AD, Fisher JO, O’Connor TM, Hughes SO. Predicting preschool children’s eating in the absence of hunger from maternal pressure to eat: A longitudinal study of low-income, Latina mothers. Appetite. 2018;120:281–286.
27. Goldthorpe J, Ali N, Calam R. Providing healthy diets for young children: the experience of parents in a UK inner city. International journal of qualitative studies on health and well-being. 2018;13(1):1490623.
28. Gomel JN, Zamora A. English- and Spanish-speaking Latina mothers’ beliefs about food, health, and mothering. Journal of immigrant and minority health. 2007;9(4):359–367.
29. Goodell LS, Johnson SL, Antono AC, Power TG, Hughes SO. Strategies Low-Income Parents Use to Overcome Their Children’s Food Refusal. Maternal and child health journal. 2017;21(1):68–76.
30. Gross RS, Brown NM, Mendelsohn AL, Katzow MW, Arana MM, Messito MJ. Maternal Stress and Infant Feeding in Hispanic Families Experiencing Poverty. Academic Pediatrics. 2021.
31. Gross RS, Mendelsohn AL, Arana MM, Messito MJ. Food Insecurity During Pregnancy and Breastfeeding by Low-Income Hispanic Mothers. Pediatrics. 2019;143(6).
32. Gross RS, Mendelsohn AL, Fierman AH, Hauser NR, Messito MJ. Maternal infant feeding behaviors and disparities in early child obesity. Childhood obesity (Print). 2014;10(2):145–152.
33. Gross RS, Mendelsohn AL, Messito MJ. Additive effects of household food insecurity during pregnancy and infancy on maternal infant feeding styles and practices. Appetite. 2018;130:20–28.
34. Gross RS, Velazco NK, Briggs RD, Racine AD. Maternal depressive symptoms and child obesity in low-income urban families. Academic pediatrics. 2013;13(4):356–363.
35. Harden J, Dickson A. Low-income mothers’ food practices with young children: A qualitative longitudinal study. Health Education Journal. 2015;74(4):381–391.
36. Harris HA, Jansen E, Mallan KM, Daniels L, Thorpe K. Concern Explaining Nonresponsive Feeding: A Study of Mothers’ and Fathers’ Response to Their Child’s Fussy Eating. Journal of nutrition education and behavior. 2018;50(8):757–764.
37. Harris HA, Jansen E, Mallan KM, Daniels L, Thorpe K. Do Dads Make a Difference? Family Feeding Dynamics and Child Fussy Eating. Journal of developmental and behavioral pediatrics: JDBP. 2018;39(5):415–423.
38. Harris HA, Staton S, Morawska A, Gallegos D, Oakes C, Thorpe K. A comparison of maternal feeding responses to child fussy eating in low-income food secure and food insecure households. Appetite. 2019;137:259–266.
39. Heinig MJ, Follett JR, Ishii KD, Kavanagh-Prochaska K, Cohen R, Panchula J. Barriers to compliance with infant-feeding recommendations among low-income women. Journal of human lactation: official journal of International Lactation Consultant Association. 2006;22(1):27–38.
40. Herman AN, Malhotra K, Wright G, Fisher JO, Whitaker RC. A qualitative study of the aspirations and challenges of low-income mothers in feeding their preschool-aged children. The international journal of behavioral nutrition and physical activity. 2012;9:132.
41. Hidalgo-Mendez J, Power TG, Fisher JO, O’Connor TM, Hughes SO. Child weight status and accuracy of perceived child weight status as predictors of Latina mothers’ feeding practices and styles. Appetite. 2019;142:104387.
42. Hodges EA, Wasser HM, Colgan BK, Bentley ME. Development of FEEDING CUES During Infancy and Toddlerhood. MCN: The American Journal of Maternal Child Nursing. 2016;41(4):244-251.
43. Hoerr SL, Hughes SO, Fisher JO, Nicklas TA, Liu Y, Shewchuk RM. Associations among parental feeding styles and children’s food intake in families with limited incomes. The International Journal of Behavioral Nutrition and Physical Activity. 2009;6.
44. Horodynski MA, Arndt MJ. ‘Eating-together’ mealtimes with African-American fathers and their toddlers. Applied Nursing Research. 2005;18(2):106–109.
45. Horodynski MA, Brophy-Herb H, Henry M, Smith KA, Weatherspoon L. Toddler feeding: expectations and experiences of low-income African American mothers. Health Education Journal. 2009;68(1):14–25.
46. Horodynski MA, Brophy-Herb HE, Martoccio TL, Contreras D, Peterson K, Shattuck M, et al. Familial psychosocial risk classes and preschooler body mass index: The moderating effect of caregiver feeding style. Appetite. 2018;123:216–224.
47. Horodynski MA, Stommel M. Nutrition education aimed at toddlers: an intervention study. Pediatric nursing. 2005;31(5):364.
48. Horodynski MA, Stommel M, Brophy-Herb H, Xie Y, Weatherspoon L. Low-income African American and non-Hispanic White mothers’ self-efficacy, “picky eater” perception, and toddler fruit and vegetable consumption. Public health nursing (Boston, Mass). 2010;27(5):408–417.
49. Hughes CC, Sherman SN, Whitaker RC. How low-income mothers with overweight preschool children make sense of obesity. Qualitative health research. 2010;20(4):465–478.
50. Hughes S, Hayes J, Sigman-Grant M, VanBrackle A. Potential Use of Food/Activity, Parenting Style, and Caregiver Feeding Style Measurement Tools with American Indian Families: A Brief Report, <Blank>: Springer Nature; 2017.
51. Hughes SO, Anderson CB, Power TG, Micheli N, Jaramillo S, Nicklas TA. Measuring feeding in low-income African-American and Hispanic parents. Appetite. 2006;46(2):215–223.
52. Hughes SO, Cross MB, Hennessy E, Tovar A, Economos CD, Power TG. Caregiver’s Feeding Styles Questionnaire. Establishing cutoff points. Appetite. 2012;58(1):393–395.
53. Hughes SO, Power TG, Beck A, Betz D, Goodell LS, Hopwood V, et al. Short-Term Effects of an Obesity Prevention Program Among Low-Income Hispanic Families With Preschoolers. Journal of nutrition education and behavior. 2020;52(3):224–239.
54. Hughes SO, Power TG, Beck AD, Betz D, Goodell LS, Hopwood V, et al. Twelve-Month Efficacy of an Obesity Prevention Program Targeting Hispanic Families With Preschoolers From Low-Income Backgrounds. Journal of nutrition education and behavior. 2021.
55. Hughes SO, Power TG, Liu Y, Sharp C, Nicklas TA. Parent emotional distress and feeding styles in low-income families. The role of parent depression and parenting stress. Appetite. 2015;92:337–342.
56. Hughes SO, Power TG, O’Connor TM, Fisher JO, Micheli NE, Papaioannou MA. Maternal feeding style and child weight status among Hispanic families with low-income levels: a longitudinal study of the direction of effects. International Journal of Behavioral Nutrition & Physical Activity. 2021;18(1):1–13.
57. Hughes SO, Power TG, O’Connor TM, Orlet Fisher J, Chen T-A. Maternal Feeding Styles and Food Parenting Practices as Predictors of Longitudinal Changes in Weight Status in Hispanic Preschoolers from Low-Income Families. Journal of obesity. 2016;2016:7201082.
58. Hughes SO, Power TG, Orlet Fisher J, Mueller S, Nicklas TA. Revisiting a neglected construct: parenting styles in a child-feeding context. Appetite. 2005;44(1):83–92.
59. Hughes SO, Power TG, Papaioannou MA, Cross MB, Nicklas TA, Hall SK, et al. Emotional climate, feeding practices, and feeding styles: an observational analysis of the dinner meal in Head Start families. The international journal of behavioral nutrition and physical activity. 2011;8:60.
60. Hughes SO, Shewchuk RM. Child temperament, parent emotions, and perceptions of the child’s feeding experience. The international journal of behavioral nutrition and physical activity. 2012;9:64.
61. Hughes SO, Shewchuk RM, Baskin ML, Nicklas TA, Qu H. Indulgent feeding style and children’s weight status in preschool. Journal of developmental and behavioral pediatrics: JDBP. 2008;29(5):403–410.
62. Hurley KM, Pepper MR, Candelaria M, Wang Y, Caulfield LE, Latta L, et al. Systematic development and validation of a theory-based questionnaire to assess toddler feeding. The Journal of nutrition. 2013;143(12):2044–2049.
63. Jansen E, Harris HA, Mallan KM, Daniels L, Thorpe K. Measurement invariance of the Feeding Practices and Structure Questionnaire-28 among a community of socioeconomically disadvantaged mothers and fathers. Appetite. 2018;120:115–122.
64. Johnson SL, Goodell LS, Williams K, Power TG, Hughes SO. Getting my child to eat the right amount. Mothers’ considerations when deciding how much food to offer their child at a meal. Appetite. 2015;88:24–32.
65. Kaiser LL, Martinez NA, Harwood JO, Garcia LC. Research and professional briefs. Child feeding strategies in low-income Latino households: focus group observations. Journal of the American Dietetic Association. 1999;99(5):601–603.
66. Kaiser LL, Melgar-Quiñonez HR, Lamp CL, Johns MC, Harwood JO. Acculturation of Mexican-American mothers influences child feeding strategies. Journal of the American Dietetic Association. 2001;101(5):542–547.
67. Kalinowski A, Krause K, Berdejo C, Harrell K, Rosenblum K, Lumeng JC. Beliefs about the Role of Parenting in Feeding and Childhood Obesity among Mothers of Lower Socioeconomic Status. Journal of Nutrition Education & Behavior. 2012;44(5):432–437.
68. Kamdar N, Hughes SO, Chan W, Power TG, Meininger J. Indirect Effects of Food Insecurity on Body Mass Index Through Feeding Style and Dietary Quality Among Low-Income Hispanic Preschoolers. Journal of nutrition education and behavior. 2019;51(7):876–884.
69. Karp SM, Lutenbacher M, Dietrich MS. The associations of psychosocial factors and infant feeding beliefs and practices of young, first time, low income mothers. Issues in comprehensive pediatric nursing. 2010;33(4):268–287.
70. Khalsa AS, Woo JG, Kharofa RY, Geraghty SR, DeWitt TG, Copeland KA. Parental intuitive eating behaviors and their association with infant feeding styles among low-income families. Eating behaviors. 2019;32:78–84.
71. Kong A, Jones BL, Fiese BH, Schiffer LA, Odoms-Young A, Kim Y, et al. Parent-child mealtime interactions in racially/ethnically diverse families with preschool-age children. Eating behaviors. 2013;14(4):451–455.
72. Kong A, Vijayasiri G, Fitzgibbon ML, Schiffer LA, Campbell RT. Confirmatory factor analysis and measurement invariance of the Child Feeding Questionnaire in low-income Hispanic and African-American mothers with preschool-age children. Appetite. 2015;90:16–22.
73. Kröller K, Warschburger P. Associations between maternal feeding style and food intake of children with a higher risk for overweight. Appetite. 2008;51(1):166–172.
74. Kugler KC, Balantekin KN, Birch LL, Savage JS. Application of the multiphase optimization strategy to a pilot study: an empirical example targeting obesity among children of low-income mothers. BMC public health. 2016;16(1):1181.
75. Liew J, Zhou Z, Perez M, Yoon M, Kim M. Parental Child-feeding in the Context of Child Temperament and Appetitive Traits: Evidence for a Biopsychosocial Process Model of Appetite Self-Regulation and Weight Status. Nutrients. 2020;12(11).
76. Lindsay AC, Sussner KM, Greaney ML, Peterson KE. Latina mothers’ beliefs and practices related to weight status, feeding, and the development of child overweight. Public health nursing (Boston, Mass). 2011;28(2):107–118.
77. Lindsay AC, Wallington SF, Lees FD, Greaney ML. Exploring How the Home Environment Influences Eating and Physical Activity Habits of Low-Income, Latino Children of Predominantly Immigrant Families: A Qualitative Study. International journal of environmental research and public health. 2018;15(5).
78. Lumeng JC, Miller AL, Appugliese D, Rosenblum K, Kaciroti N. Picky eating, pressuring feeding, and growth in toddlers. Appetite. 2018;123:299–305.
79. Maher S, Lopez P, McKee MD, Deen D, Fornari A, Fletcher J, et al. Evaluation of health educator consults in primary care. Health Education. 2010;110(3):209–224.
80. Malika NM, Hayman LW, Jr., Miller AL, Lee HJ, Lumeng JC. Low-income women’s conceptualizations of food craving and food addiction. Eating behaviors. 2015;18:25–29.
81. McCurdy K, Gorman KS, Kisler T, Metallinos-Katsaras E. Associations between family food behaviors, maternal depression, and child weight among low-income children. Appetite. 2014;79:97–105.
82. Melgar-Quiñonez HR, Kaiser LL. Relationship of child-feeding practices to overweight in low-income Mexican-American preschool-aged children. Journal of the American Dietetic Association. 2004;104(7):1110–1119.
83. Messito MJ, Katzow MW, Mendelsohn AL, Gross RS. Starting Early Program Impacts on Feeding at Infant 10 Months Age: A Randomized Controlled Trial. Childhood Obesity. 2020;16:S-4.
84. Moore AM, Clair-Michaud M, Melanson KJ, Tovar A. A Pilot Feasibility Study to Improve Food Parenting Practices. American journal of health behavior. 2018;42(2):61–70.
85. Morrison H, Power TG, Nicklas T, Hughes SO. Exploring the effects of maternal eating patterns on maternal feeding and child eating. Appetite. 2013;63:77–83.
86. Mosli RH, Lumeng JC, Kaciroti N, Peterson KE, Rosenblum K, Baylin A, et al. Higher weight status of only and last-born children. Maternal feeding and child eating behaviors as underlying processes among 4-8 year olds. Appetite. 2015;92:167–172.
87. Murashima M, Hoerr SL, Hughes SO, Kaplowitz SA. Feeding behaviors of low-income mothers: directive control relates to a lower BMI in children, and a nondirective control relates to a healthier diet in preschoolers. The American journal of clinical nutrition. 2012;95(5):1031–1037.
88. Musaad SMA, Speirs KE, Hayes JT, Mobley AR, Fitzgerald N, Jones BL, et al. The impact of environmental, parental and child factors on health-related behaviors among low-income children. Appetite. 2017;112:260–271.
89. Na M, Jomaa L, Eagleton SG, Savage JS. Head Start Parents With or Without Food Insecurity and With Lower Food Resource Management Skills Use Less Positive Feeding Practices in Preschool-Age Children. Journal of Nutrition. 2021;151(5):1294–1301.
90. Nix RL, Francis LA, Feinberg ME, Gill S, Jones DE, Hostetler ML, et al. Improving Toddlers’ Healthy Eating Habits and Self-regulation: A Randomized Controlled Trial. Pediatrics. 2021;147(1):1–8.
91. Omar MA, Coleman G, Hoerr S. Healthy eating for rural low-income toddlers: Caregivers’ perceptions. Journal of Community Health Nursing. 2001;18(2):93–106.
92. Ontai LL, Sitnick SL, Shilts MK, Townsend MS. My child at mealtime: A visually enhanced self-assessment of feeding styles for low-income parents of preschoolers. Appetite. 2016;99:76–81.
93. Orr CJ, Ben-Davies M, Ravanbakht SN, Yin HS, Sanders LM, Rothman RL, et al. Parental Feeding Beliefs and Practices and Household Food Insecurity in Infancy. Academic pediatrics. 2019;19(1):80–89.
94. Orr CJ, Ravanbakht S, Flower KB, Yin HS, Rothman RL, Sanders LM, et al. Associations Between Food Insecurity and Parental Feeding Behaviors of Toddlers. Academic pediatrics. 2020.
95. Orrell-Valente JK, Hill LG, Brechwald WA, Dodge KA, Pettit GS, Bates JE. “Just three more bites”: an observational analysis of parents’ socialization of children’s eating at mealtime. Appetite. 2007;48(1):37–45.
96. Papaioannou MA, Cross MB, Power TG, Liu Y, Qu H, Shewchuk RM, et al. Feeding style differences in food parenting practices associated with fruit and vegetable intake in children from low-income families. Journal of nutrition education and behavior. 2013;45(6):643–651.
97. Parkes A, Sweeting H, Young R, Wight D. Does parenting help to explain socioeconomic inequalities in children’s body mass index trajectories? Longitudinal analysis using the Growing Up in Scotland study. Journal of Epidemiology and Community Health. 2016;70(9):868–873.
98. Perez M, Ohrt TK, Bruening AB, Taylor AB, Liew J, Kroon Van Diest AMW, et al. Measurement equivalence of child feeding and eating measures across gender, ethnicity, and household food security. BMC obesity. 2018;5:17.
99. Pesch MH, Appugliese DP, Kaciroti N, Rosenblum KL, Miller AL, Lumeng JC. Maternal encouragement and discouragement: Differences by food type and child weight status. Appetite. 2016;101:15–22.
100. Pesch MH, Daniel AR, Miller AL, Rosenblum KL, Appugliese DP, Lumeng JC, et al. Feeding styles among mothers of low-income children identified using a person-centered multi-method approach. Appetite. 2020;146:104509.
101. Pesch MH, Miller AL, Appugliese DP, Rosenblum KL, Lumeng JC. Affective tone of mothers’ statements to restrict their children’s eating. Appetite. 2016;103:165–70.
102. Petrunoff NA, Wilkenfeld RL, King LA, Flood VM. ‘Treats’, ‘sometimes foods’, ‘junk’: a qualitative study exploring ‘extra foods’ with parents of young children. Public health nutrition. 2014;17(5):979–986.
103. Pineros-Leano M, Tabb K, Liechty J, Castañeda Y, Williams M. Feeding decision-making among first generation Latinas living in non-metropolitan and small metro areas. PloS one. 2019;14(3):e0213442.
104. Power TG, Beck AD, Fisher JO, Micheli N, O’Connor TM, Hughes SO. Observations of Maternal Feeding Practices and Styles and Young Children’s Obesity Risk: A Longitudinal Study of Hispanic Mothers with Low Incomes. Childhood Obesity. 2021;17(1):16–25.
105. Power TG, Hughes SO, Goodell LS, Johnson SL, Duran JAJ, Williams K, et al. Feeding practices of low-income mothers: how do they compare to current recommendations? The international journal of behavioral nutrition and physical activity. 2015;12:34.
106. Power TG, O’Connor TM, Orlet Fisher J, Hughes SO. Obesity Risk in Children: The Role of Acculturation in the Feeding Practices and Styles of Low-Income Hispanic Families. Childhood obesity (Print). 2015;11(6):715–721.
107. Power TG, Silva Garcia K, Beck AD, Goodell LS, Johnson SL, Hughes SO. Observed and self-reported assessments of caregivers’ feeding styles: Variable- and person-centered approaches for examining relationships with children’s eating behaviors. Appetite. 2018;130:174–183.
108. Powers SW, Chamberlin LA, van Schaick KB, Sherman SN, Whitaker RC. Maternal feeding strategies, child eating behaviors, and child BMI in low-income African-American preschoolers. Obesity (Silver Spring, Md). 2006;14(11):2026–2033.
109. Redsell SA, Atkinson P, Nathan D, Siriwardena AN, Swift JA, Glazebrook C. Parents’ beliefs about appropriate infant size, growth and feeding behaviour: implications for the prevention of childhood obesity. BMC public health. 2010;10:711.
110. Reicks M, Randall JL, Haynes BJ. Factors affecting consumption of fruits and vegetables by low-income families. Journal of the American Dietetic Association. 1994;94(11):1309–1311.
111. Sacco LM, Bentley ME, Carby-Shields K, Borja JB, Goldman BD. Assessment of infant feeding styles among low-income African-American mothers: comparing reported and observed behaviors. Appetite. 2007;49(1):131–140.
112. Santos JL, Kain J, Dominguez-Vásquez P, Lera L, Galván M, Corvalán C, et al. Maternal anthropometry and feeding behavior toward preschool children: association with childhood body mass index in an observational study of Chilean families. The international journal of behavioral nutrition and physical activity. 2009;6:93.
113. Savage JS, Birch LL. WIC mothers’ depressive symptoms are associated with greater use of feeding to soothe, regardless of perceived child negativity. Pediatric obesity. 2017;12(2):155–162.
114. Searle B-RE, Harris HA, Thorpe K, Jansen E. What children bring to the table: The association of temperament and child fussy eating with maternal and paternal mealtime structure. Appetite. 2020;151:104680.
115. Shriver LH, Hamm EW, Buehler CA. Predictors of fruit and vegetable intake in low-income and racially diverse preschoolers: does parental feeding style matter? Journal of Public Health (09431853). 2019;27(4):407–418.
116. Silva Garcia K, Power TG, Beck AD, Fisher JO, Goodell LS, Johnson SL, et al. Stability in the feeding practices and styles of low-income mothers: questionnaire and observational analyses. The international journal of behavioral nutrition and physical activity. 2018;15(1):28.
117. Silva Garcia K, Power TG, Fisher JO, O’Connor TM, Hughes SO. Latina mothers’ influences on child appetite regulation. Appetite. 2016;103:200–207.
118. Skala K, Chuang R-J, Evans A, Hedberg A-M, Dave J, Sharma S. Ethnic differences in the home food environment and parental food practices among families of low-income Hispanic and African-American preschoolers. Journal of immigrant and minority health. 2012;14(6):1014–1022.
119. Slusser W, Erausquin JT, Prelip M, Fischer H, Cumberland WG, Frankel F, et al. Nutrition knowledge and behaviours of low-income Latino parents of preschoolers: Associations with nutrition-related parenting practices. Early Child Development and Care. 2012;182(8):1041–1055.
120. Sparks MA, Radnitz CL. Child disinhibition, parent restriction, and child body mass index in low-income preschool families. Journal of nutrition education and behavior. 2013;45(1):82–85.
121. Sun A, Cheng J, Bui Q, Liang Y, Ng T, Chen J-L. Home-Based and Technology-Centered Childhood Obesity Prevention for Chinese Mothers With Preschool-Aged Children. Journal of transcultural nursing: official journal of the Transcultural Nursing Society. 2017;28(6):616–624.
122. Tan CC, Walczak M, Roach E, Lumeng JC, Miller AL. Longitudinal associations between eating and drinking engagement during mealtime and eating in the absence of hunger in low income toddlers. Appetite. 2018;130:29–34.
123. Tartaglia J, McIntosh M, Jancey J, Scott J, Begley A. Exploring Feeding Practices and Food Literacy in Parents with Young Children from Disadvantaged Areas. International journal of environmental research and public health. 2021;18(4).
124. Thompson AL, Adair LS, Bentley ME. Pressuring and restrictive feeding styles influence infant feeding and size among a low-income African-American sample. Obesity (Silver Spring, Md). 2013;21(3):562–571.
125. Thompson AL, Mendez MA, Borja JB, Adair LS, Zimmer CR, Bentley ME. Development and validation of the Infant Feeding Style Questionnaire. Appetite. 2009;53(2):210–221.
126. Trappmann JL, Jimenez EY, Keane PC, Cohen DA, Davis SM. Cross-Sectional Relationships Between Household Food Insecurity and Child BMI, Feeding Behaviors, and Public Assistance Utilization Among Head Start Children From Predominantly Hispanic and American Indian Communities in the CHILE Study. Journal of hunger & environmental nutrition. 2015;10(4):439–455.
127. Ventura AK, Gromis JC, Lohse B. Feeding practices and styles used by a diverse sample of low-income parents of preschool-age children. Journal of nutrition education and behavior. 2010;42(4):242–249.
128. Weatherspoon LJ, Venkatesh S, Horodynski MA, Stommel M, Brophy-Herb HE. Food patterns and mealtime behaviors in low-income mothers and toddlers. Journal of community health nursing. 2013;30(1):1–15.
129. Wehrly SE, Bonilla C, Perez M, Liew J. Controlling parental feeding practices and child body composition in ethnically and economically diverse preschool children. Appetite. 2014;73:163–171.
130. Worobey J, Borrelli A, Espinosa C, Worobey HS. Feeding Practices of Mothers from Varied Income and Racial/Ethnic Groups. Early child development and care. 2013;183(11):1661–1668.
131. Zhou Z, Liew J, Yeh Y-C, Perez M. Appetitive Traits and Weight in Children: Evidence for Parents’ Controlling Feeding Practices as Mediating Mechanisms. The Journal of genetic psychology. 2020;181(1):1–13.
